# Self-Incompatibility in Apricot: Identifying Pollination Requirements to Optimize Fruit Production

**DOI:** 10.3390/plants11152019

**Published:** 2022-08-03

**Authors:** Sara Herrera, Jorge Lora, José I. Hormaza, Javier Rodrigo

**Affiliations:** 1 Departamento de Ciencia Vegetal, Centro de Investigación y Tecnología Agroalimentaria de Aragón (CITA), Avda. Montañana 930, 50059 Zaragoza, Spain; sherreral@aragon.es; 2 Instituto Agroalimentario de Aragón-IA2, CITA-Universidad de Zaragoza, 50013 Zaragoza, Spain; 3 Subtropical Fruit Crops Department, Instituto de Hortofruticultura Subtropical y Mediterránea La Mayora (IHSM La Mayora-CSIC-UMA), 29750 Algarrobo-Costa, Spain; jlora@eelm.csic.es (J.L.); ihormaza@eelm.csic.es (J.I.H.)

**Keywords:** apricot, pollen tube, pollination, *Prunus armeniaca*, *S*-alleles, self-incompatibility

## Abstract

In recent years, an important renewal of apricot cultivars is taking place worldwide, with the introduction of many new releases. Self-incompatible genotypes tolerant to the sharka disease caused by the plum pox virus (PPV), which can severely reduce fruit production and quality, are being used as parents in most breeding programs. As a result, the self-incompatibility trait present in most of those accessions can be transmitted to the offspring, leading to the release of new self-incompatible cultivars. This situation can considerably affect apricot management, since pollination requirements were traditionally not considered in this crop and information is lacking for many cultivars. Thus, the objective of this work was to determine the pollination requirements of a group of new apricot cultivars by molecular identification of the *S*-alleles through PCR amplification of *RNase* and *SFB* regions with different primer combinations. The *S*-genotype of 66 apricot cultivars is reported, 41 for the first time. Forty-nine cultivars were considered self-compatible and 12 self-incompatible, which were allocated in their corresponding incompatibility groups. Additionally, the available information was reviewed and added to the new results obtained, resulting in a compilation of the pollination requirements of 235 apricot cultivars. This information will allow an efficient selection of parents in apricot breeding programs, the proper design of new orchards, and the identification and solution of production problems associated with a lack of fruit set in established orchards. The diversity at the *S*-locus observed in the cultivars developed in breeding programs indicates a possible genetic bottleneck due to the use of a reduced number of parents.

## 1. Introduction

Apricot (*Prunus armeniaca* L.) belongs to the genus *Prunus* in the Rosaceae family. This crop was domesticated in different domestication events ca. 2000–3000 years ago in Central Asia, which is considered the center of origin of the crop [1,2]. Later, apricot was introduced into the Mediterranean Basin from the Caucasus, a secondary center of diversification [3]. Nowadays, apricot world production has reached 3.7 million tons [4] and it is considered one of the most economically important fruit crops in temperate regions [5].

Apricot cultivars have been traditionally classified into six eco-geographical groups according to their geographical origin: Central Asian, East Chinese, North Chinese, Dzhungar-Zailij, Irano-Caucasian, and European [6]. Apricot cultivars from the Central Asian, which is the oldest and most diverse, the Dzhungar-Zailij, and the Iranian-Caucasian groups, are mostly self-incompatible. Commercial cultivars of Europe, North America, South Africa, and Australia are mainly self-compatible and belong to the European group [7], which has two main gene pools: Continental Europe and Mediterranean Europe [8].

Gametophytic Self-Incompatibility (GSI) is a mechanism to prevent self-fertilization and promote outcrossing present in the Rosaceae [9]. It is controlled by a multiallelic locus named *S* that contains several genes involved in the pollen-pistil recognition. The *S-RNase* gene encodes pistil-expressed glycoproteins with ribonuclease activity that act as highly selective cytotoxins that cause rejection of pollen when its *S*-allele is the same as either of the two *S*-alleles expressed in the pistil [10,11]. Consequently, pollen tube growth is arrested in the style preventing fertilization [12]. The *SFB* gene, which codifies an F- box protein, is specifically expressed in the pollen, and determines the pollen allele specificity [13]. Nowadays, there are self-incompatible and self-compatible apricot cultivars, as in other *Prunus* species such as almond (*Prunus dulcis*), sweet cherry (*Prunus avium*), Japanese plum (*Prunus salicina*), European plum (*Prunus domestica*) and sour cherry (*Prunus cerasus*) [7]. In recent years, the system seems to be more complicated, with the report of different modifier genes involved in the incompatibility response [14,15].

The pollination requirements of cultivars can be established by evaluation of fruit set after self- and cross-pollination under field conditions [16,17], by pollen tube growth observations in self- and cross-pollinated flowers under fluorescence microscopy with the advantage of avoiding failures caused by adverse weather conditions [18,19,20,21,22], and by molecular techniques based on PCR and sequencing approaches of the *S*-locus [23]. In apricot, 33 *S*-alleles (*S*_1_ to *S*_20_, *S*_22_ to *S*_30_, *S*_52_, *S*_53_, *S_v_*, and *S_x_*) have been identified so far, including one allele linked to self-compatibility (*S_c_*) [16,24,25,26,27].

In recent years, an important renewal of apricot cultivars is taking place worldwide, with the introduction of many new releases in response to productive and industrial changes in the crop [28]. The main objectives of breeding programs for the development of new commercial cultivars include sharka-tolerance/resistance, climate adaptability and improved organoleptic properties fruit such as firmness, skin and fresh color, and aroma [29]. The sharka disease was reported for the first time in plum around 1915 and, since then, it has become the most economically important virus disease of *Prunus* species [30]. PPV is usually transmitted by aphids, and probably spread by the propagation of infected plant material [31]. Sharka disease causes discoloration on leaves, petals and fruits, severely reducing fruit production and quality [31].

Self-(in)compatibility and inter-(in)compatibility relationships in apricot have been characterized in traditional and local cultivars from different regions such as China [26], Hungary [32], Morocco [33], North America and Spain [16,21,34], Tunisia [17,35], and Turkey [32,36,37]. Although self-(in)compatibility of new apricot releases are evaluated in some public breeding programs (‘Centro de Edafología y Biología Aplicada del Segura’ (CEBAS-CSIC) in Murcia [38,39], and the ‘Instituto Valenciano de Investigaciones Agrarias’ (IVIA) in Valencia [40], both in Spain; ‘Institut National de la Recherche Agronomique’ (INRA) in France [41,42,43]; University of Bologna and University of Milan in Italy [44]; ‘Agricultural Research Service’ in Parlier, CA, and Rutgers University in New Brunswick, NJ, in the USA [45]), the pollination requirements of many cultivars are still unknown.

In this work, we evaluate the hypothesis that a significant proportion of new apricot cultivars are self-incompatible and that knowing the incompatibility relationships between cultivars is needed to select appropriate pollinating cultivars in the design of orchards. For this purpose, the *S*-genotype of 66 apricot cultivars was analyzed; of them, 49 were self-compatible and the other 12 were self-incompatible. The results allowed allocating the self-incompatible cultivars in their corresponding incompatibility groups according to their *S*-alleles. In addition, a compilation of the available *S*-genotype data was carried out to evaluate the distribution of the *S*-alleles in the main apricot cultivars grown worldwide and to assess their current genetic diversity.

## 2. Results

### 2.1. S-Alleles and Incompatibility Groups

**Figure 1:**
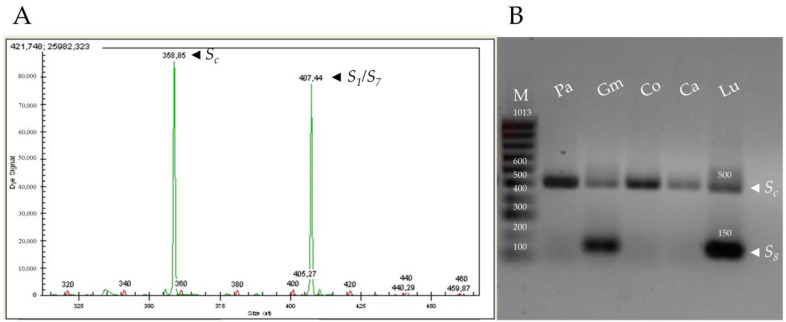
Size of the PCR amplification fragments using different primer pair combinations for the identification of *S*-alleles. (**A**) Gene analyzer output for the SRc-(F/R) primers showing the size of the two amplified fragments of the *RNase* first intron region corresponding to the *S*-alleles *S_c_* (358 bp, left) and *S*_1_/*S*_7_ (408 bp, right) in apricot cv. ‘Rojo Pasión’. (**B**) PCR amplification with the AprFBC8-(F/R) primers for identifying *S_c_*- and *S*_8_-alleles in five apricot cultivars (Pa: ‘Paviot’, Gm: ‘Gönci Magyarkajszi’, Co: ‘Corbato’, Ca: ‘Canino’, and Lu: ‘Luizet’). M: 100 bp DNA Ladder.

PCR analysis based on the amplification of *RNase* and *SFB* genes allowed identifying the *S*-genotype in 66 apricot cultivars, 41 of them reported for the first time (Table 1 and Table 2). Firstly, the combination of the primers SRc-F/SRc-R amplified the first intron of the *RNase*, and the different alleles were classified according to the sizes of the fragments previously established by Vilanova et al. [25] (Figure 1A; Supplementary Table S1). To our knowledge, the sequence of the first intron has only been reported in the *S*_1_, *S*_2_, *S*_4_, *S*_6_, *S*_7_ and *S_c_*-alleles [22]. The PruC2/PruC4R primer combination amplified the second intron and was used to differentiate the *S*_6_ and *S*_9_ alleles in 15 genotypes, whose sequences were also previously reported [21,46]. The SHLM1 and SHLM2 primer combination amplified a fragment of 650 bp in 17 cultivars, indicating the presence of the *S*_1_-allele. A 413 bp fragment, that corresponds to the *S*_7_-allele, was only detected in ‘Charisma’ and ‘Ninfa’ using the primer pair SHLM3/SHLM4 [22]. 

**Figure 2:**
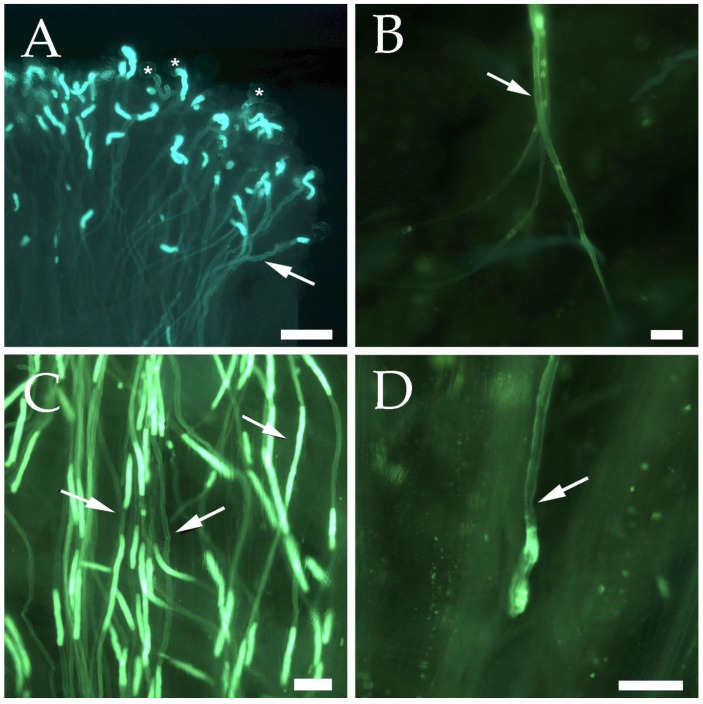
Pollen germination and pollen tube growth in self-pollinated apricot flowers observed under the microscope. In Gametophytic Self-Incompatibility (GSI), both compatible and incompatible pollen grains germinate on the stigma. The pollen grain carries one of the two *S*-alleles of the original genotype. In self-incompatible cultivars, if the *S*-allele of the pollen grain matches one of the two *S*-alleles of the pistil, pollen tube growth is inhibited in the middle part of the style. (**A**) Pollen grains (*) germinating at the stigma surface with pollen tubes emerging towards the style the style (arrow) in the self-compatible cultivar ‘Water’. (**B**) Pollen tubes (arrow) reaching the base of the style (down) in the self-compatible cultivar ‘Water’. (**C**) Pollen tubes (arrows) growing along the style in the self-compatible cultivar ‘Water’. (**D**) Pollen tube (arrow) arrested in the middle part of the style in the self-incompatible cultivar ’Samourai’. Aniline blue staining for callose of squash preparations. Scale bars = 100 µm.

PCR amplification of the *S_c_*- and *S*_8_-alleles using the primers designed based on the first and second introns of the *RNase* sequence produced identical size fragments [52]. Thus, the AprFBC8-F/AprFBC8-R primers for the *SFB* region were used for distinguishing both *S*-alleles [32]. This primer combination amplified a fragment of 150 bp, allowing to identify the *S*_8_-haplotype in 3 cultivars, whereas a 500 bp fragment from V2 region was amplified to characterize the *S_c_*-allele in 47 cultivars (Figure 1B; Supplementary Table S1). However, the primers AprFBC8-F/AprFBC8-R did not produce amplification fragments in two cultivars (‘Samourai’ and ‘Water’). Thus, controlled pollinations were carried out to differentiate the *S_c_*- and *S*_8-_alleles in these two cultivars. All self-pollinated pistils showed germinated pollen grains on the stigma in both cultivars (Figure 2A). ‘Water’ behaved as self-compatible since all the self-pollinated pistils (*n* = 17) showed pollen tubes arriving to the base of the style (Figure 2B). In ‘Samourai’, pollen tubes reached the upper third of the style in all the self-pollinated pistils analyzed (Figure 2C), but pollen tube growth ceased in the middle part of the style, forming a callose tip (Figure 2D), with a mean percentage of style traveled by the pollen tubes of 62.5% (*n* = 10). Thus, ‘Samourai’ was considered as self-incompatible. In addition, all the examined pistils cross-pollinated with ‘Katy’ showed pollen tubes at the base of the style in ‘Water’ (*n* = 20) and ‘Samourai’ (*n* = 5).

The *S*-genotype of eleven cultivars, in which a unique allele could be previously characterized [21], has been completed using several primer combinations. The specific primers SHLM1 and SHLM2 allowed to identify the *S*_1_-allele in five cultivars (‘Farely’ (*S*_1_*S*_9_), ‘Harcot’ (*S*_1_*S*_4_), ‘Megatea’ (*S*_1_*S*_9_), ‘Monster Cot’ (*S*_1_*S*_9_), and ‘Priabel’ (*S*_1_*S*_9_)). Primers PruC2 and PruC4R enabled the identification of a second *S*-allele, *S*_6_ in ‘Pandora’ (*S*_2_*S*_6_), ‘Muñoz’ (*S*_2_*S*_6_), ‘Fartoly’ (*S_c_S*_6_), ‘Lady Cot’ (*S_c_S*_6_), and *S*_9_ in ‘Tadeo’ (*S_c_S*_9_). The genotype of ‘Luizet’ (*S_c_S*_8_) was identified using the AprFBC8-F/AprFBC8-R primer combination. Additionally, the presence of the *S_c_*- and *S*_8_-alleles was confirmed in 19 cultivars using the AprFBC8-F and AprFBC8-R primers. In these 19 cultivars, observations of pollen tube growth in pollination experiments were previously used to establish self(in)compatibility; the *S_c_*-allele was assigned to self-compatible cultivars, and the *S*_8_-allele to self-incompatible accessions [21]. Moreover, the results allowed to confirm the *S*-genotype of eight cultivars; ‘Búlida’ (*S*_5_*S_c_*) [16], ‘Canino’ (*S*_2_*S_c_*) [34], ‘Cebas Red’ (*S_c_*) [53], ‘Gönci magyarkajszi’ (*S*_8_*S _c_*) [52], ‘Harcot’ (*S*_1_*S*_4_) [49], ‘Katy’ (*S*_1_*S*_2_) [55], ‘Ninfa’ (*S*_7_*S_c_*) [16] and ‘Tilton’ (*S*_2_*S_c_*) [47]; a single *S_c_*-allele amplification was obtained in ‘Mirlo rojo’ and ‘Primorosa’, which were reported previously as self-compatible (*S_c_S_c_*) [53,54]. 

In order to establish the compatibility relationships among cultivars, the 12 self-incompatible cultivars were allocated in their corresponding incompatibility groups according to their *S*-genotypes. In five cultivars (´Dama Toronja´, ´Tornado´, ´Vitillo´, ´Fuego´, ´Mogador´), self-(in)compatibility could not be established by their *S*-genotype because only one allele other than *S_c_* could be identified and, consequently, they were considered unclassified (Table 1). 

### 2.2. Diversity in the S-Locus Region

The *S*-genotypes of 235 apricot accessions, including the 60 cultivars analyzed herein in which two *S*-alleles could be identified together with 175 accessions previously reported (Table 1 and Table 2; Supplementary Table S2) were used to assess the diversity and differentiation at the gametophytic self-incompatibility *S*-locus. 

**Figure 3:**
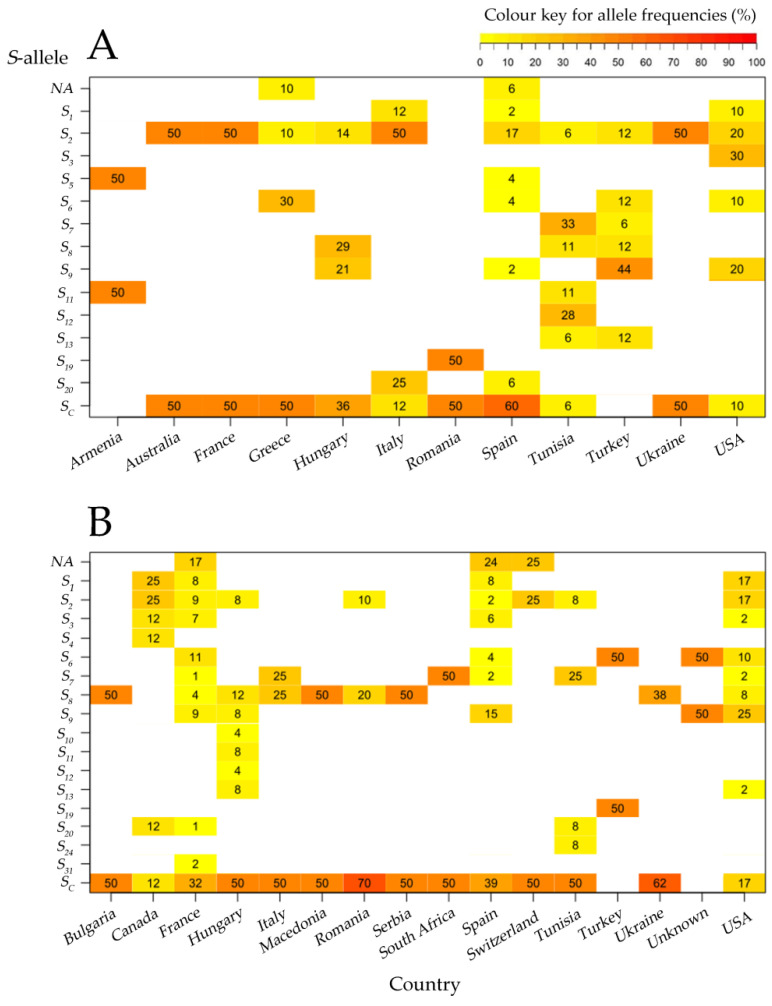
Heatmaps of allele frequencies in traditional cultivars (**A**) and cultivars from breeding programs (**B**) for each country of origin using “PopGenReport” v. 3.0.4 R package. Cell color indicates the proportion of the total number of alleles, and the numbers within a cell show the percentage of the number of alleles in each country. The frequency of each *S*-allele was calculated in each country within each group of accessions, showing statistically significant relationships (*p* < 0.05) between *S*-alleles and countries in the groups of both traditional cultivars and releases from breeding programs.

Fourteen *S*-alleles in 32 *S*-locus combinations were identified within 70 traditional apricot accessions, whereas 36 *S*-genotype combinations with 17 *S*-alleles were found in the group of 157 releases from breeding programs (Table 3 and Table 4). Both groups of accessions showed the same value of the average number of alleles per country (N_a_ = 4). Thirteen *S*-alleles were present in both groups (Figure 3). However, the alleles *S*_4_, *S*_10_, *S*_24_, and *S*_31_ were only identified in the commercial cultivars ‘Ezzine´ (*S*_24_), ‘Harmat´(*S*_10_), ‘Harcot´(*S*_4_), ‘Cow-1´(*S*_31_), and ‘Cow-2´(*S*_31_), and the *S*_5_-allele was only found in the traditional cultivars ‘Búlida’ and ‘Velázquez’, from Spain, and ‘Shalakh’, from Armenia.

In both traditional cultivars and cultivars released from breeding programs, *S_c_* was the most frequent *S*-allele as it was found in 38 and 101 cultivars, respectively. The *S_c_*-allele was not detected in Armenian and Turkish germplasm but was present in more than 50% of the genotypes from Australia, France, Greece, Romania, Spain, and Ukraine (Figure 3A; Supplementary Table S3). A similar trend was observed in cultivars from breeding programs, except for cultivars from North America (Canada and the USA), in which the *S_c_*-allele appeared in less than 20% of the cultivars (Figure 3B; Supplementary Table S4).

In traditional cultivars, *S*_2_ was the second most frequent *S*-allele (*n* = 24), followed by *S*_9_ (*n* = 13), *S*_6_ and *S*_8_ (*n* = 8), S_7_ (*n* = 7), *S*_12_ and *S*_20_ (*n* = 5), *S*_1_, *S*_3_, *S*_5_, *S*_11_, and *S*_13_ (*n* = 3), and *S*_19_ (*n* = 1). In cultivars from breeding programs, *S*_9_ was the second most frequent *S*-allele (*n* = 35) followed by *S*_2_ (*n* = 26), *S*_1_ (*n* = 24), *S*_6_ (*n* = 23), *S*_8_ (*n* = 21), *S*_3_ (*n* = 14), *S*_7_ (*n* = 8), *S*_13_ and *S*_20_ (*n* = 3), *S*_11_ and *S*_31_ (*n* = 2) and *S*_4_, *S*_10_, *S*_12_, *S*_19_, *S*_24_ (*n* = 1) (Supplementary Tables S2–S4).

A higher percentage of single alleles was observed in cultivars from breeding programs compared to traditional cultivars: 17% in France, 24% in Spain, and 25% in Switzerland for the cultivars from breeding programs compared to 10% in Greece and 6% in Spain for the traditional cultivars (Figure 3).

Regarding the allele frequencies, the chi-squared tests showed a statistically significant relationship (*p* < 0.05) between *S*-alleles and countries in the groups of both traditional cultivars and releases from breeding programs (Supplementary Tables S3 and S4).

The existence of private alleles, *S*-alleles that are found only in a single population, was used as an indicator of genetic differentiation between both groups. Three alleles (P_a_) were found in the group of traditional cultivars. *S*_3_ was only found in ‘Perfection’ and ‘Sun Glo’ from the USA, *S*_12_ in ‘Bedri Ahmar’, ‘Bouthani Ben Friha’, ‘Oud Rhayem’, and ‘Oud Hmida’, from Tunisia, and *S*_19_ in ‘Mari de Cenad’ from Romania. In the group of releases from breeding programs, seven private alleles were identified, three of them, *S*_10_, *S*_11_ and *S*_12_, present in Hungarian accessions (‘Harmat’, ‘Korai zamatos’, and ‘Voski’).

Although the number of genotypes in the group of cultivars from breeding programs was nearly twice than those in the group of traditional cultivars, the same value of average allelic richness (A_r_ = 1.70) was found in both groups. Comparisons of traditional cultivars with cultivars from breeding programs revealed a slight loss of diversity for the *S*-locus in Hungarian, Tunisian, and Turkish modern cultivars. On the contrary, higher allelic richness in cultivars from breeding programs than in traditional cultivars were observed in France, Italy, and Spain.

## 3. Discussion

### 3.1. Self- and Cross-Incompatibility in Apricot

The *S*-genotype of 66 apricot cultivars was reported herein, 41 for the first time. A total of 49 cultivars were characterized as self-compatible since their genotype contained the *S_c_*-allele, which is associated with self-compatibility [56]. However, the self-compatibility of two cultivars was determined through controlled pollinations due to mismatching of PCR primers resulting in no amplification. When self(in)compatibility cannot be determined by identifying the *S*-genotype, laboratory pollination experiments have proven to be an accurate method because they avoid weather-related failures under field conditions [23,57,58].

Here, the *S*-genotype has been characterized in 11 cultivars [’Pandora’ and ‘Muñoz’ (*S*_2_*S*_6_), ‘Farely’, ‘Megatea’, ‘Monster Cot’, and ‘Priabel’ (*S*_1_*S*_9_), ‘Harcot’ (*S*_1_*S*_4_), ‘Fartoly’ and ‘Ladycot’ (*S*_6_*S_c_*), ‘Luizet’ (*S*_8_*S_c_*), and ‘Tadeo’ (*S*_9_*S_c_*)], in which only one *S*-allele could be previously identified [21], probably by the mismatching of PCR primers or preferential amplification of the detected allele. The use of specific primers for the *S*_1_-allele [22] allowed the identification of this allele in 15 cultivars for the first time. The *S_c_*-allele was confirmed in 19 cultivars in which self-compatibility was previously assessed by cross-pollinations [21]. AprFBC8-(F/R) primers allowed to distinguish between the *S_c_*- and *S*_8_-alleles, since an insertion of 358 bp in the *SFB* gene causes a loss of the incompatibility that has been observed in the *SFB_c_* gene but not in the sequence of *SFB*_8_ [52,56]. Therefore, the cultivars carrying the *S*_8_-allele but not the *S_c_*-allele, such as ’Sweet Cot’, might be considered as self-incompatible. A single *S_c_*-allele was identified in 21 genotypes. As this allele is associated with self-compatibility [56], these cultivars could present homozygosity, as it has been considered in previous reports for some cultivars that have been characterized as *S_c_S_c_* [16,25,34,35,47,52]. Our results agree with previous reports of the *S*-genotype for ‘Búlida’ [16], ‘Cebas Red’ [53], ‘Canino’ [34], ‘Gönci magyarkajszi’ [52], ‘Harcot’ [49], ‘Katy’ [55], ‘Ninfa’ [16] and ‘Tilton’ [47]. According to Egea et al. [59], ‘Rojo Pasión’ resulted from a cross between ‘Orange Red’ (*S*_6_*S*_9_ [16,21]) and ‘Currot’ (*S_c_S_c_* [34]). However, our results for the *S*-genotype (*S*_1_*S_c_*) differ from this pedigree. Additionally, our results for three cultivars, ‘Alba’, ‘Corbato’, and ‘Tadeo’, differ from the *S*-genotype previously reported [16].

According to their *S*-allele composition, 12 self-incompatible cultivars were allocated in their corresponding cross-incompatibility groups together with the 77 self-incompatible cultivars previously analyzed [16,21,22,24,32,34,35,47,48,49,50]. To date, a total of thirty-five incompatibility groups (I to XXXV) have been described in apricot [17,21,22,32,35,48]. Self-incompatible cultivars within the same incompatibility group have the same *S*-genotype and are genetically incompatible with each other. On the other hand, cultivars from different incompatibility groups are inter-compatible, since at least one of the *S*-alleles of their genotype is different [60]. Although lower yields have been related to semi-compatible pollinizers (when one *S*-allele is identical and the other differs) in Japanese plum and sweet cherry [61,62,63], there is no information on the effects of semi-compatibility in apricot. In addition, 16 cultivars are included in group 0 since no other cultivars with the same *S*-genotype have been reported until now. Thus, the cultivars from group 0 as well as the self-compatible cultivars could act as universal pollinizers. This could be highly valuable information since the knowledge of incompatibility relationships aims to help breeders to choose parental genotypes for breeding programs and fruit growers to select compatible pollinizers coincident at flowering time.

### 3.2. Current Genetic Diversity at the S-Locus

To provide an overview of the genetic diversity at the *S*-locus of currently grown apricot cultivars, results herein have been combined with previous results [16,21,22,24,25,32,34,35,39,47,48,49,50,52,54,55] to analyze the frequency and distribution of the *S*-alleles. A total of 33 *S*-alleles (*S*_1_ to *S*_20_, *S*_22_ to *S*_30_, *S*_52_, *S*_53_, *S_c_*, *S_v_*, and *S_x_*) have been described in apricot cultivars [16,24,25,26,27]. Fourteen *S*-alleles were detected within the group of 70 traditional cultivars from Armenia, Australia, France, Greece, Hungary, Italy, Romania, Spain, Tunisia, Turkey, Ukraine, and the USA, whereas 17 *S*-alleles were identified in the group of cultivars from breeding programs, reflecting lower *S*-allele diversity in the group of traditional cultivars. Twenty *S*-alleles (20) were reported in a group of 67 cultivars from Europe and North America [16], but fewer *S*-alleles were found when local accessions or landraces were studied separately in Tunisia [17,35], Turkey [32,36,37], and Morocco [33].

Four *S*-alleles (*S*_10_, *S*_24_, *S*_31_ and *S*_4_) were exclusively found in the group of cultivars from breeding programs, which could be related to the use of landraces instead of commercial cultivars in some breeding programs. Two of these *S*-alleles showed a clear relationship with a specific breeding program: *S*_24_ was found in ‘Ezzine’ from INRAT (Tunisia) [64], and *S*_31_ was found in ‘Cow-1′ and ‘Cow-2′ from INRA (France) [65]; *S*_4_ was present in the North American cultivar ‘Harcot’ [49]. Although this cultivar has been used as a parental genotype in several breeding programs to introduce Sharka-resistance to new releases [29], the *S*_4_-allele has not been found in any recent releases. On the other hand, the *S*_5_-allele has been previously reported only in some traditional cultivars from Spain and Armenia. Our results differed in the *S*-genotype of the Spanish landrace ‘Corbato’ in which this allele has been previously reported, (*S_c_* vs. *S*_2_*S*_5_ [16]). The presence of the *S*_5_-allele in this cultivar, as well as in other traditional cultivars from Spain and Armenia, has been reported by Muñoz-Sanz et al. [16]. They suggested that the presence of the *S*_5_-allele in those populations could be the result of a connection between Southern-Spanish accessions with the Armenian and Eastern-Turkish accessions; in addition, they suggested that Moroccan accessions are a part of the Southwest-Mediterranean apricot diffusion route.

Traditionally, the main marker for self-compatibility in apricot has been *S_c_* [49]. For this reason, one of the main objectives of breeding programs has been to introduce this *S*-allele into new releases [11]. Our results showed that *S_c_* was the most frequent *S*-allele in both the groups of traditional and cultivars from breeding programs. Although we did not detect the *S_c_*-allele in traditional cultivars from Armenia and Turkey, previous reports found some self-compatible Turkish cultivars carrying this *S*-allele [32,37]. The *S_c_*-allele might have evolved in Southeastern Turkey as a result of a pollen-part mutation within *SFB*_8_, causing the pollen with the mutated *S*_8_ haplotype to be self-compatible [52]. Subsequently, it is hypothesized that the allele *S_c_* was disseminated to the Mediterranean Basin since the cultivars from Central Asia, the center of origin of apricot, are self-incompatible [52,66]. The *S_c_*-allele was present in cultivars from breeding programs in most countries except for Turkey, probably due to the low number of new releases from this country studied in this work. However, the low presence of the *S_c_*-allele in North American cultivars (the USA and Canada) is probably due to the fact that most of these cultivars are self-incompatible [28,67].

The presence of just one allele has been observed in a high number of cultivars, mostly cultivars released from breeding programs, mainly as genotype *S_c_*- (Supplementary Table S2) that would presumably correspond to *S_c_S_c_*. Homozygote cultivars can arise as a result of self- and cross-pollinations with self-compatible parents in breeding programs. Although this genotype has been frequently found in apricot cultivars from most European countries [16,25,34,52], results herein as well as those from previous studies could not confirm homozygosity since sequencing would be needed [22].

The distribution of *S*-alleles varied considerably between countries, with a significant association (*p* < 0.05) between *S*-alleles and geographical origin, both in the group of traditional accessions and in the group of breeding program releases. Within traditional cultivars, the *S*_3_-allele was found exclusively in North American cultivars [16]. The *S*_12_-allele was only found in four cultivars from Tunisia [35], being one of the most frequent *S*-alleles in this country [17]. The *S*_19_-allele was only found in one cultivar from Romania, ‘Mari de Cenad’, despite having been associated with Hungarian and Turkish local apricots [32]. Muñoz-Sanz et al. [16] reported that the *S*_19_-allele found in this cultivar could be *S*_20_; however, further sequencing would be required to confirm this.

Alleles *S*_10_ to *S*_14_ have Armenian origin [24] but they have also been described in cultivars from Eastern Europe [32], Morocco [33], Tunisia [17,35] and Turkey [32,37]. Our results showed three of these alleles (*S*_10_, *S*_11_, and *S*_12_) in three Hungarian traditional cultivars, ‘Harmat’, ‘Korai zamatos’, and ‘Voski’.

Thus, in addition to providing useful information to know the self-compatibility of cultivars, *S*-genotyping can be a valuable tool in elucidating the evolution and dissemination of the crop.

### 3.3. Self-Compatibility and Diversity

Self-compatibility has been described as a cause of loss of genetic diversity since it promotes inbreeding [68]. Self-incompatibility not only reduces inbreeding by preventing self-fertilization but also reduces mating between close relatives, ensuring the exchange of genetic material [69]. It has been suggested that the loss of genetic diversity affecting the *S*-locus is due to crop dissemination [16]. In fact, a bottleneck has been observed in apricot diversity as a consequence of the domestication and diffusion of the apricot throughout the history of the crop [8]. Additionally, a decrease in genetic diversity from the eastern (Iran-Caucasian area) to the south-western (North Mediterranean Basin and South Mediterranean Basin areas) distribution of the crop has been detected, analyzing local cultivars from Algeria, France, Italy, Morocco, Spain, Tunisia, and Turkey [70]. A lower number of *S*-alleles were found in accessions from Moroccan oases as compared to the whole allele pool in this country, probably due to the pressure to increase production and self-compatibility in the genotypes, allowing a higher level of endogamy [33]. A similar situation was described in landraces from Central Europe [52].

Apricots belonging to the European group have been traditionally considered to be self-compatible [71]. However, the number of self-incompatible commercial cultivars in the European group increased rapidly over the last two decades due to the use of self-incompatible North American cultivars as parentals in breeding programs [21,28]. Recent studies show that about half of the new releases are self-compatible: 51.1% (47 of 92) [21] and 49.6% (61 of 123) [20].

Recently, additional sources of self-compatibility have been described in apricot in addition to the *S_c_*-allele. Thus, some works have reported the existence of an additional mutation in the *M*-locus not linked to the *S*-locus, which causes a loss of pollen *S*-activity [16,55,56,72]. Pollen-part mutations (PPMs) in the *M*-locus were mapped at the distal end of chromosome three in ‘Canino’ (called m [72]) and in ‘Katy’ (called m’ [55]). Muñoz-Sanz et al. [73] proposed the *ParMDO* gene as a relevant gene involved in pollen part SI function. In order to optimize the screening of self-compatible genotypes, a new useful method based on both loci has been recently developed [74].

Although a clear trend towards releasing self-compatible cultivars is shown, our results exhibited similar allelic richness values in both groups, traditional cultivars, and cultivars from breeding programs. A clear differentiation between apricot landraces and cultivars from breeding programs was recently revealed using SSR markers, showing an unexpected higher diversity in cultivars from breeding programs, which was related to the use of North American genotypes as parentals [75]. Our results showed a slight loss of diversity for the *S*-locus in Hungarian, Tunisian, and Turkish cultivars comparing landraces with releases from breeding programs. However, this situation was not observed in the countries included in the North Mediterranean Basin group such as France, Italy, and Spain. This could be due to the higher number of new releases from breeding programs of these countries.

Although there is no evidence of a reduction in the diversity at the *S*-locus in cultivars developed in breeding programs, results herein suggests that the use of a reduced number of parents in breeding programs can lead to a genetic bottleneck.

## 4. Materials and Methods

### 4.1. Plant Material

Young leaves from 66 apricot cultivars, including traditional cultivars (landraces and local selections) and releases from breeding programs of several origins (Table 1 and Table 2), were collected in spring from germplasm collections and orchards in Spain. Moreover, flowers were collected from two cultivars (‘Samourai’ and ‘Water’) for pollination experiments to establish the self-(in)compatibility by microscopic observations. The apricot accessions analyzed originated from 12 countries: Armenia, Australia, France, Greece, Hungary, Italy, Romania, Spain, Tunisia, Turkey, Ukraine, and the USA.

### 4.2. DNA Extraction and S-Allele Identification

Genomic DNA of each sample was extracted using DNeasy Plant Mini Kit (Qiagen, Hilden, Germany) according to Hormaza [76] and quantified by NanoDrop™ ND-1000 spectrophotometer (Bio-Science, Budapest, Hungary).

The *S*-genotype of each cultivar was identified through PCR amplification of *RNase* and *SFB* regions with different primer combinations (Table 5) [23]. The first intron of the *S-RNase* gene was amplified with the fluorescently labeled primer combination SRc-(F/R) [25,77]. PCR amplifications were carried out in 15 µL reaction volumes, containing 10× NH_4_ Reaction Buffer, 25 mM MgCl_2_, 2.5 mM of each dNTP, 10 µM of each primer, 100 ng of genomic DNA and 0.5 U of BioTaq^TM^ DNA polymerase (Bioline, London, UK). The temperature profile used had an initial step of 3 min at 94 °C, 35 cycles of 1 min at 94 °C, 1 min at 55 °C and 3 min at 72 °C, and a final step of 5 min at 72 °C. The amplified fragments were analyzed in a CEQ^TM^ 8000 capillary electrophoresis DNA analysis system (Beckman Coulter, Fullerton, CA, the USA) and classified according to Vilanova et al. [25] and Herrera et al. [21].

Because two pairs of alleles, *S*_6_/*S*_9_ and *S*_1_/*S*_7_, showed similar fragment sizes, specific primers based on the second intron of the *RNase* were used to distinguish between them. For the identification of the *S*_6_- and *S*_9_-alleles, the PruC2/PruC4R primer combination designed from *P. avium S-RNase*-cDNA sequences [78] was used to differentiate both alleles in 15 genotypes. Specific primers SHLM1/SHLM2 and SHLM3/SHLM4 were required to distinguish between *S*_1_ and *S*_7_, respectively [22]. PCR reactions were carried out according to Vilanova et al. [25], but with the addition of 10 cycles and using 55 °C of annealing temperature [79]. The amplified fragments were separated on 1% (*w*/*v*) agarose gels and the DNA bands were visualized using the nucleic acid stain SYBR Green (Thermo Fisher Scientific, St Leon-Rot, Germany). For the identification of the *S*_1_-allele, the specific primers SHLM1 and SHLM2 were used following the protocol described by Herrera et al. [22] for *Taq* DNA polymerase (Qiagen, Hilden, Germany). Primers SHLM3 and SHLM4 were used for *S*_7_-allele identification. PCR reactions were performed with Phusion^®^ High-Fidelity DNA Polymerase (Thermo Fisher Scientific, St Leon-Rot, Germany) according to Herrera et al. [22].

Since PCR amplification of the *S_c_*- and *S*_8_-alleles using the primers SRc-(F/R) provides a fragment of similar size [24,56], the specific primers AprFBC8-F and AprFBC8-R, designed based on the V2 and HVb variable region of *SFB* gene, were used to distinguish between both alleles [32]. The PCR amplifications were carried out using the program previously described by Halász et al. [32].

### 4.3. Pollination Experiments

In cultivars ‘Samourai’ and ‘Water’, no amplification was produced with the primers AprFBC8-F/AprFBC8-R. Thus, controlled pollinations were carried out in these two cultivars to differentiate the *S_c_*- and *S*_8_-alleles. Self-(in)compatibility was established by laboratory-controlled pollinations and the observation of pollen tube growth under fluorescence microscopy [23]. Self-pollinations were carried out in the two cultivars. Pollen of the cultivar ‘Katy’, known as universal pollinizer for apricot [55], was used to pollinize another set of flowers of each cultivar as control.

Flowers from each cultivar were collected at the balloon stage one day before anthesis and emasculated to avoid self-pollination. Pistils were placed on wet florist foam and maintained at laboratory temperature. After 24 h, a group of 20–25 flowers were hand-pollinated with the help of a paintbrush for each self- and cross-pollination [80]. Pollen was obtained from flowers at the same balloon stage by removing and drying the anthers at laboratory temperature during 24 h. Pollen grains were then sieved by using a fine mesh (0.26 mm) and used immediately or frozen at −20 °C until further use. Seventy-two hours after pollination, pistils were fixed in ethanol (95%)/acetic acid (3:1, *v*/*v*) during 24 h, and conserved at 4 °C in 75% ethanol. After hand pollinations, pollen viability was evaluated. Pollen from each pollen donor was scattered on a solidified pollen germination medium [81] and pollen germination was observed under the microscope after 24 h. Pollen grains were considered viable when the length of the growing pollen tube was higher than the pollen grain diameter.

For histochemical preparations, the fixed pistils were washed three times for 1 h with distilled water and left in 5% sodium sulphite in distillated water at 4 °C for 24 h. Then, they were autoclaved at 1 kg/cm^2^ during 10 min in sodium sulphite to soften the tissues [82]. Pistils were squashed and stained with 0.1% (*v*/*v*) aniline blue in 0.1N K_3_PO_4_ [83] to observe callose. Examination of pollen tube growth was carried out by fluorescence microscopy using by a Leica DM2500 microscope (Cambridge, UK) with UV epifluorescence using 340–380 bandpass and 425 longpass filters.

Pollen tube behavior was observed in at least 10 pistils in each self-pollination. Cultivars were considered as self-incompatible when pollen tube growth was arrested along the style in most self-pollinated pistils. On the other hand, when the pollen tube reached the base of the style in most self-pollinations, the cultivars were considered as self-compatible.

### 4.4. S-Allele Diversity Analysis

In order to analyze the *S*-allele genetic diversity, the *S-RNase* genotypes of the 60 cultivars identified herein were compiled with those of the 175 cultivars previously reported (Table 1 and Table 2; Supplementary Table S2). Cultivars were first classified into two groups according to the pedigree origin of the accessions, traditional, and releases from breeding programs. Statistical analyses were performed using the R programming environment (R Core Team, 2022, version 4.1.0, Vienna, Austria). The *S*-genetic profiles were stored in a csv file which was converted into a matrix of allelic frequencies stored in a genind class with the “loci2genind” function using the R package “pegas” version 1.0–1 [84]. Missing data (<0.1%) were replaced with the mean frequency of the corresponding allele, which avoids adding artefactual between-group differentiation [85].

The number of alleles (N_a_), allelic richness (A_r_), and private alleles (P_a_) were calculated for all countries on the traditional and cultivars from breeding programs using the adegenet 2.1.3 [85], and PopGenReport 3.0.4 [86] packages. Additionally, the frequency of each *S*-allele was calculated in each country within each group and the results were plotted as a heatmap with the R package PopGenReport version 3.0.4 [86]. To analyze the relationship between the *S*-alleles and their distribution by country, a contingency table of absolute frequencies of alleles by country was created and a chi-square test was performed with the “chisq.test” function using the R package “stats” v. 4.1.2. Due to the low number of observations in some countries, a Monte Carlo simulation with 2000 replicates was indicated.

## 5. Conclusions

Results reveal that a significant proportion of new apricot releases are self-incompatible and, therefore, require cross-pollination to produce fruit. Knowing the incompatibility relationships between cultivars will help breeders to select suitable parental genotypes in crosses. This information, combined with the flowering dates in each geographical area, will allow the selection of appropriate inter-compatible pollinizers for self-incompatible cultivars in the design of new orchards. The identification of the *S*-alleles, in addition to the determination of the pollination requirements of the cultivars, can elucidate missing gaps in the evolution, domestication, and dissemination of the crop. The diversity at the *S*-locus observed in the cultivars developed in breeding programs indicates a possible genetic bottleneck due to the use of a reduced number of parents in breeding programs.

## Figures and Tables

**Table 1:** Incompatibility group (I.G.) and *S*-genotype of 103 apricot cultivars.

I.G. (*S*-Genotype)	Cultivars Analyzed in This Study	Cultivars Analyzed in Previous Studies
I (*S*_1_*S*_2_)		AC1 [21], Castleton [16], Farmingdale [47], Giovanniello [47], Goldrich [48], Hargrand [48], Lambertin-1 [48]
II (*S*_8_*S*_9_)		Ceglédi óriás [24], Cologlu [32], Ligeti óriás [24], Perlecot [21], Pinkcot [21], Szegedi M. [16]
III (*S*_2_*S*_6_)	Muñoz ^b^, Pandora ^b^	ASF0401 [21], Avirine (Bergarouge) [21], Moniqui [49]
IV (*S*_2_*S*_7_)		Ouardi [35], Priana [34]
V (*S*_2_*S*_8_)	Sweet Cot ^d^ [21]	Alyanak [32], Holly Cot [21]
VIII (*S*_6_*S*_9_)	Apribang (ASF0405) ^a^	ASF0402 [21], Cataloglu [32], Cheyenne [22], Feria Cot [21], Flashcot [50], JNP [21], Ninja [50], Orangered [16,21], Soganci [32], Stark Early Orange [16,21], Sunny Cot [21], Wonder Cot [21]
X (*S*_7_*S*_12_)		Bedri Ahmar [35], Oud Rhayem [35]
XI (*S*_9_*S*_13_)		Haci Haliloglu [32], Kabaasi [32]
XII (*S*_11_*S*_13_)		Voski [24]
XIII (*S*_6_*S*_19_)		Levent [32]
XV (*S*_7_*S*_8_)		Oueld El Oud [35]
XVI (*S*_7_*S*_11_)		Bouk Ahmed [35], Hamidi [35]
XVII (*S*_8_*S*_12_)		Adedi Ahmar [35]
XVIII (*S*_1_*S*_3_)	IPS23214 ^a^, Monred ^a^	Cooper Cot [21], Perfection [48]
XIX (*S*_2_*S*_3_)		Mayacot [21], Sun Glo [49]
XX (*S*_2_*S*_9_)		Goldstrike 02 [21], Hasanbey [32], Magic Cot [21]
XXI (*S*_3_*S*_8_)	Samourai ^a,c^	Lilly Cot [21], Spring Blush [21]
XXII (*S*_3_*S*_9_)		Almadulce [21], Flodea [21], Henderson [16,21], Kosmos [22], Tsunami [50]
XXIII (*S*_7_*S*_9_)		Goldbar [21], Kurukabuk [32]
XXVI (*S*_1_*S*_6_)		Primaya [22]
XXV (*S*_1_*S*_9_)	Farely ^b^, Megatea ^b^, Monster Cot ^b^, Priabel ^b^	Almater [50], Aurora [50], Medaga [50]
XXVI (*S*_6_*S*_8_)		Robada [50]
Group 0	Harcot ^b^ (*S*_1_*S*_4_) [49]	Bouthani Ben Friha (*S*_12_*S*_13_) [35], Cow-1 (*S*_1_*S*_31_) [16], Cow-2 (*S*_20_*S*_31_) [16], Estrella (*S*_1_*S*_7_) [51], Harlayne (*S*_3_*S*_20_) [16], Harmat (*S*_10_*S*_11_) [24], Korai zamatos (*S*_12_*S*_13_) [24], Mariem (*S*_7_*S*_20_) [16], Martinet (*S*_2_*S*_2_) [16], Oud Hmida (*S*_2_*S*_12_) [35], Perla (*S*_2_*S*_20_) [16], Portici (*S*_2_*S*_20_) [16], Shalakh (Erevani) (*S*_5_*S*_11_) [16], Velázquez (*S*_5_*S*_20_) [16]
Unclassified		
*S*_1_	Dama taronja ^a^, Tornado ^a^, Vitillo ^a^	IBCOT 18-2 [50]
*S*_2_	Fuego ^a^	Cyrano [22], IBCOT 29-5 [50], Veecot [21]
*S*_3_	Mogador ^a^	Colorado [21], Mikado [22]
*S*_6_		Stella [21]
*S*_8_		Vanilla Cot [21]
*S*_9_		Goldstrike 01 [21]

^a^ *S-RNase* genotypes first reported in this study; ^b^ *S*-genotype completed in cultivars in which previously only one allele could be identified [21]; ^c^ *S_c_*/*S*_8_ allele identified using fluorescence microscopy; ^d^ *S_c_*/*S*_8_ allele confirmed using the primers AprFBC8-F/AprFBC8-R.

**Table 2:** *S*-genotype of 153 self-compatible apricot cultivars.

*S*-Genotype	Cultivars Analyzed in This Study	Cultivars Analyzed in Previous Studies
*S*_1_ *S*_c_	Big Red ^a^, Dama Rosa ^a^, Flavorcot ^a^, Rojo Pasión ^a^, Rubissia ^a^, Water ^a,c^	Mauricio [34]
*S*_2_ *S*_c_	Bergecot ^d^ [21], Canino ^d^ [21,34], Harval ^a^, Justo Cot ^a^, Paviot ^d^ [21], Primidi ^d^ [21], Tilton [47]	Berdejo [21,50], Bergeron [52], Budapest [52], Dulcinea [16], Galta Vermella Valenciana [16], Kalao [22], Konservnyi Pozdnii [52], Mamaia [52], Mandulakajszi [52], Mediva [21,50], Peñaflor 02 [21,50], Pepito del Rubio [49,50], Rakovszky [52], Regibus [22], Roxana [52], Rózsakajszi [16], Sandy cot [21,50], Trevatt [16]
*S*_3_ *S*_c_	Rubista ^d^ [21]	Pricia [21], Rambo [22]
*S*_5_ *S*_c_	Búlida [16]	
*S*_6_ *S*_c_	Fartoly ^b,d^ [21], Ladycot ^b,d^ [21], Medflo ^d^ [21], Mediabel ^d^ [21]	Aprix20 [21,50], Aprix9 [21,50], Bebecou [47], Faralia [21,50], Farlis [21,50], Lito [16]
*S*_7_ *S*_c_	Charisma ^d^ [21], Ninfa [16]	Beliana [34], Sayeb [35]
*S*_8_ *S*_c_	Gönci Magyarkajszi [52], Luizet ^b,d^ [21]	Andornaktályai magyarkajszi [52], Cacansko zlato [52], Callatis [52], Crvena ungarska [52], Darunec malahoyeva [52], Effect [52], Kâsna ungarska [52], Krimskyi Amur [52], Nagygyümölcsû magyarkajszi [52], Nikitskyi [52], Paksi magyarkajszi [52], Pisana [52], Venus [52]
*S*_9_ *S*_c_	Alba ^a^, Aprisweet (ASF0409) ^a^, Micaelo ^a^, Tadeo ^b^ [21]	AC2 [21,50], Ceglédi arany [52], Ceglédi bíborkajski [52], Flopria [21], Lido [22], Tom Cot [21]
*S*_13_ *S*_c_		Modesto [24]
*S*_19_ *S*_c_		Mari de Cenad [16]
*S*_20_ *S*_c_		Cristalí [16], Gavatxet [16]
*S*_24_ *S*_c_		Ezzine [16]
*S_c_S*_c_		Ananasnyi ciurpinskii [52], Asli [35], Borsi-féle kései rózsa [52], Ceglédi kedves [52], Currot [34], GaltaRoja [16], Gandía [16], Ginesta [25], Grandir [47], Manrí [16], NJA-8 [52], Nyujtó Ferenc emléke [52], Palabras [16], Palau [25], Pannónia [52], Pasinok [52], Patterson [47], Raki [35], Rojo Carlet [16], Sirena [52], Sulmona [52], Tirynthos [16], Xirivello [16], Zaposdolye [52]
*S*_c_	Aprix 116 ^a^, Cebas Red [53], Cocot ^a^, Corbato ^d^ [21], Delice cot ^d^ [21], Fantasme ^a^, Farhial ^d^ [21], IPS21512 ^a^, IPS2712 ^a^, Laguna ^a^, Merino ^a^, Mirlo anaranjado ^d^ [21,54], Mirlo blanco ^d^ [21,54], Mirlo Rojo [54], Mitger ^d^ [21], Orange rubis ^a^, Precoz de Tirynthos ^a^, Primorosa [53], Soledane ^d^ [21], Tardorange ^a^, Valorange ^a^	Aprix 33 [21,50], ASF0404 (Apriqueen) [21,50], Dorada [22], Faclo [21,50], Farbaly [21,50], Farbela [21,50], Fardao [21,50], Farfia [21,50], Farius [21,50], IBCOT 13-12 [50], IPS16121 [50], Kioto [50], Memphis [22], Milord [22], Murciana [22], Oscar [22], Playa cot [21,50], Rouge cot [21], Rubely [50], Sherpa [22] Swired [21,50]
*S*_1_ *S*_2_	Katy [55]	Lorna [21], Palsteyn [21]
*S*_2_ *S*_9_		Victor 1 [21]
*S*_1_		IPS20390 [50], Rubilis [50]
*S*_3_		Golden Sweet [21]

^a^ *S-RNase* genotype first reported in this study; ^b^ *S*-genotype completed in cultivars in which previously only one allele could be identified [21]; ^c^ *S_c_*/*S*_8_ allele identified using fluorescence microscopy; ^d^ *S_c_*/*S*_8_ allele confirmed using the primers AprFBC8-F/AprFBC8-R.

**Table 3:** Genetic parameters of apricot traditional cultivars including landraces and local selections. Number of cultivars, number of alleles (N_a_), allelic richness (A_r_), and number of private alleles (P_a_) for each country of origin. SD: standard deviation; SE: standard error.

Country	Number of Cultivars	Number of Alleles (N_a_)	Allelic Richness (A_r_)	Number of Private Alleles (P_a_)
Armenia	1	2	1.67	-
Australia	1	2	1.67	-
France	2	2	1.57	-
Greece	5	3	1.67	-
Hungary	7	4	1.75	-
Italy	4	4	1.70	-
Romania	1	2	1.67	1 (*S*_19_)
Spain	26	7	1.61	-
Tunisia	9	7	1.80	1 (*S*_12_)
Turkey	8	6	1.77	-
Ukraine	1	2	1.67	-
The USA	5	6	1.84	1 (*S*_3_)
**Total**	70	47		3
**Mean ± SD**		4 ± 2	1.70 ± 0.08	
**SE**		0.63	0.02	

**Table 4:** Genetic parameters of apricot cultivars released from breeding programs. Number of cultivars, number of alleles (N_a_), allelic richness (A_r_), and number of private alleles (P_a_) for each country of origin. SD: standard deviation; SE: standard error.

Country	Number of Cultivars	Number of Alleles (N_a_)	Allelic Richness (A_r_)	Number of Private Alleles (P_a_)
Bulgaria	1	2	1.67	-
Canada	4	6	1.87	1 (*S*_4_)
France	58	10	1.83	1 (*S*_31_)
Hungary	13	8	1.72	3 (*S*_10_, *S*_11_, *S*_12_)
Italy	2	3	1.71	-
Macedonia	1	2	1.67	-
Romania	5	3	1.48	-
Serbia	1	2	1.67	-
South Africa	1	2	1.67	-
Spain	33	7	1.76	-
Switzerland	2	2	1.71	-
Tunisia	6	5	1.70	1 (*S*_24_)
Turkey	1	2	1.67	1 (*S*_19_)
Ukraine	4	2	1.50	-
Unknown	1	2	1.67	-
The USA	24	9	1.84	-
**Total**	157	67		7
**Mean ± SD**		4 ± 3.05	1.70 ± 0.10	
**SE**		0.76	0.03	

**Table 5:** SSR primers used in this study for the identification of *S*-alleles in apricot (*Prunus armeniaca*).

Amplified Region	Name	Specificity	Primer Sequence (5′ → 3′)	Reference
***S-RNase* 1st intron**				
	SRc-(F/R)		F: CTCGCTTTCCTTGTTCTTGC	[77]
			R: GGCCATTGTTGCACCCCTTG	
***S-RNase* 2nd intron**				
	Pru-C2/C4R		F: CTTTGGCCAAGTAATTATTCAAACC	[78]
			R: GGATGTGGTACGATTGAAGCG	
	SHLM1/SHLM2	*S*_1_ *-allele*	F: GGTGGAGGTGATAAGGTAGCC	[22]
			R: GGCTGCATAAGGAAGCTGTAGG	
	SHLM3/SHLM4	*S*_7_ *-allele*	F: TATATCTTACTCTTTGGC	[22]
			R: CACTATGATAATGTGTATG	
***SFB***				
	AprFBC8-(F/R)		F: CATGGAAAAAGCTGACTTATGG	[32]
			R: GCCTCTAATGTCATCTACTCTTAG	

## Data Availability

Not applicable.

## Supplementary Materials

The following supporting information can be downloaded at: https://www.mdpi.com/article/10.3390/plants11152019/s1, Table S1: Sizes of PCR fragment amplification using five primer pair combinations for the identification of *S*-alleles and *S*-genotype of the analyzed apricot accessions; Table S2: Origin and *S*-genotype of the 235 analyzed apricot accessions; Table S3: Contingency table of absolute allele frequencies by country in apricot traditional cultivars including landraces and local selections and Pearson’s chi-squared test with simulated *p*-value (based on 2000 replicates); Table S4: Contingency table of absolute allele frequencies by country in apricot cultivars released from breeding programs and Pearson’s chi-squared test with simulated *p*-value (based on 2000 replicates).
